# Splenectomy induces biochemical remission and regeneration in experimental murine autoimmune hepatitis

**DOI:** 10.1186/s40001-022-00933-3

**Published:** 2022-12-10

**Authors:** Janine Dywicki, Laura Elisa Buitrago-Molina, Fatih Noyan, Jerome Schlue, Konstantinos Iordanidis, Michael P. Manns, Heiner Wedemeyer, Elmar Jaeckel, Matthias Hardtke-Wolenski

**Affiliations:** 1 Department of Gastroenterology, Hepatology and Endocrinology, Hannover Medical School, Hannover, Germany; 2 Institute of Pathology, Hannover Medical School, Hannover, Germany; 3 Department of Liver Transplantation, Multi Organ Transplant Program, University Health Network, University of Toronto, Toronto, Canada; 4 Institute of Medical Microbiology, University Hospital Essen, University Duisburg-Essen, Essen, Germany

**Keywords:** Autoimmune hepatitis, Transaminases, Splenectomy, Regeneration, Biochemical remission

## Abstract

Autoimmune hepatitis (AIH) is a chronic immune-mediated inflammatory liver disease. It is known that AIH originates not from the spleen but from the liver itself. Nonetheless, most details of the etiology and pathophysiology are unknown. We induced experimental murine AIH (emAIH) in NOD/Ltj mice by single administration of a replication-deficient adenovirus and performed splenectomy during late-stage disease. Biochemical disease remission occurred, which was characterized by improvement in transaminase levels. The causes of this remission included a shift in the transcriptomic signature of serum proteins toward regeneration. At the cellular level, there was a marked decrease in activated CD8^+^ T cells and an increase in intrahepatic regulatory T cells (Tregs). Here, intrahepatic Treg numbers correlated with biochemical remission. Notably, an imbalance in the T-cell/B-cell ratio was observed, with a disproportionate increase in total B cells. In summary, intrahepatic increases in Tregs, biochemical remission, and regeneration could be induced by splenectomy in the late stage of emAIH.

## Introduction

Autoimmune hepatitis (AIH) is a chronic immune-mediated inflammatory liver disease with an unclear pathogenesis. It is known that AIH does not originate in the spleen but in the liver itself [1–4]. Furthermore, genetic predisposition and environmental triggers play important roles in the development of AIH [5–8]. Moreover, AIH is generally thought to result from T lymphocyte-mediated destruction of hepatocytes. Similarly, experimental murine AIH (emAIH) can be induced by the transfer of CD4^+^ T cells alone [9].

This explains why the current therapy, which has been in use for decades, is corticosteroid administration with or without azathioprine [10, 11]. This therapy has the well-known side effects of steroids, and there up to 15% of nonresponses are due to medication intolerance, incomplete response, and treatment failure [12–14]. The alternatives studied in small local cohorts of patients include infliximab (anti-TNF-a), rituximab (anti-CD20) and low-dose IL-2 treatment. Recently, the latter therapies have shown good results in emAIH [15, 16]. Additionally, in the 1960s, some patients with chronic hepatitis were treated by splenectomy with reasonable success rates [17, 18]. Notably, this was done before a possible differential diagnosis of viral hepatitis.

The pathology of AIH is characterized histologically by interface hepatitis and the presence of plasma cells and serologically by the presence of elevated serum transaminase, immunoglobulin G, and autoantibody levels [10, 11]. Nonetheless, the roles of B cells, characteristic autoantibodies and the spleen in the course and progression of disease are uncertain.

Therefore, we induced emAIH in NOD/Ltj mice and then performed splenectomy during the late stage of disease at week 12 [9]. We measured the biochemical remission of the disease and analyzed the transcriptomic signature of serum proteins. At the cellular level, flow cytometry and intrahepatic immunofluorescence microscopy were performed to specifically examine at regulatory T cells.

## Materials and methods

### Ethics statement

The animal care and experiments were performed in accordance with the institutional and national guidelines. All animal experiments were performed according to protocols approved by the animal welfare commission of the Hannover Medical School and local ethics animal review board (Lower Saxony State Office for Consumer Protection and Food Safety, Oldenburg, Germany).

### Mice

NOD/Ltj mice were bred and maintained under specific pathogen-free conditions at the central animal facility of the Hannover Medical School (Hannover, Germany). Mice were injected intravenously with a total of 4 × 10^9^ infectious particles containing an adenovirus expressing formiminotransferase cyclodeaminase (Ad-FTCD) in PBS. Mice (*n* = 5) were splenectomized 12 weeks later in the late stage of disease [9]. All mice (*n* = 10) were sacrificed 18 weeks after infection, and then all analyses were performed.

### Adenovirus construction

Ad-FTCD was generated as described previously [9]. In brief, FTCD was amplified from human liver cells. The constructs were fused to the Ad transfer vector pShuttle-CMV (Stratagene). By homologous recombination, this shuttle vector was recombined with pAdEasy-1, which carries deletions in the E1 and E3 regions (Stratagene). The generated adenovirus genome can be amplified only within the HEK 293 packaging cell line, complementing the essential regions. Purification of recombinant adenovirus was performed using a cesium chloride gradient, and the adenoviral stocks were quantified using an Adeno-X™ Rapid Titer Kit (Clontech).

### Flow cytometry

To obtain single-cell suspension, livers were minced by a gentleMACS Dissociator (Miltenyi Biotec, Bergisch Gladbach). Then, red blood cells were lysed (eBioscience™ 1X RBC Lysis Buffer, Thermo Fisher Scientific) and IHLs (intrahepatic lymphocytes) were separated using a 40%/70% Percoll (GE Healthcare) gradient. Subsequently, lymphocytes were stained with anti-CD3, anti-CD4, anti-CD8, anti-Ki-67, anti-B220, anti-Foxp3, and anti-CD62L antibodies. All image acquisitions were performed with an LSRII SORP interfaced with DIVA software (BD Biosciences) as described previously [16, 19].

### Serum analysis

Blood samples were collected via the retroorbital route before sacrificing the mice. Aspartate aminotransferase (AST) and alanine transaminase (ALT) levels were determined by photometric enzyme activity assays with an Olympus AU400 Chemistry Analyzer using serum as described previously [9, 20].

### Histology and immunohistology

Murine liver tissue was fixed in formalin and embedded in paraffin or only embedded in Tissue-Tek® O.C.T.™ Compound (Sakura) for cryosections (8 µm). Paraffin-embedded sections (5 µm) were prepared for hematoxylin and eosin (HE) staining and analyzed with an AxioImagerM1 using AxioVision 4.8 software (Zeiss). The sections were further examined in a blinded manner by a pathologist using the approved modified hepatitis activity index (mHAI) for autoimmune hepatitis as described previously [5, 21]. Immunofluorescence microscopy was performed as previously described [11–13]. Briefly, 4 µm cryosections were fixed with acetone, rehydrated, blocked, stained with anti-CD4, anti-CD8, anti-Foxp3 and DAPI and analyzed with an AxioImagerM1 as above.

### Protein detection in serum

Proteins were measured using an Olink® MOUSE EXPLORATORY panel* (Olink Proteomics AB, Uppsala, Sweden) according to the manufacturer’s instructions as described previously [1, 22]. The proximity extension assay (PEA) technology used for the Olink protocol has been well described [23] and allows for 92 analytes to be analyzed simultaneously. Briefly, pairs of oligonucleotide-labeled antibody probes bind to their targeted protein, and if the two probes are brought in close proximity, the oligonucleotides will hybridize in a pairwise manner. The addition of DNA polymerase leads to a proximity-dependent DNA polymerization event, which generates a unique PCR target sequence. The resulting DNA sequence is subsequently detected and quantified using a microfluidic real-time PCR instrument (Biomark HD, Fluidigm). The data are then quality-controlled and normalized using an internal extension control and an interplate control to adjust for intra- and inter-run variations. The final assay read-out is presented in Normalized Protein eXpression (NPX) values, which is an arbitrary unit on a log2-scale in which a high value corresponds to high protein expression. All assay validation data (detection limits, intra- and interassay precision data, etc.) are available on the manufacturer’s website (www.olink.com).

### Real-time PCR using fluidigm technology

RNA was isolated, cDNA was preamplified, and quantitative RT-PCR was performed as described previously [2, 19]. Normalization of the Ct was performed by subtracting the mean values of the housekeeping genes *glyceraldehyde-3-phosphate dehydrogenase (Gapdh)* and a*ctin beta (Actb)* from those of genes of interest. Heatmaps and PCA plots of the − delta Ct values were created via Qlucore software (*p* < 0.05 and *q* < 0.2).

### Statistics

Unpaired Student 2-tailed *t* tests, correlation analysis, principal component analysis (PCA) and heatmap analysis were performed using GraphPad Prism version 7.00 for Mac and Qlucore Omics Explorer 3.5. * significant difference with *p* ≤ 0.05; ** very significant difference; *p* ≤ 0.01; *p* > 0.05 was considered to be nonsignificant (ns).

## Results

### Splenectomy during the late stage of disease shifts serum proteins toward regeneration

**Fig. 1:**
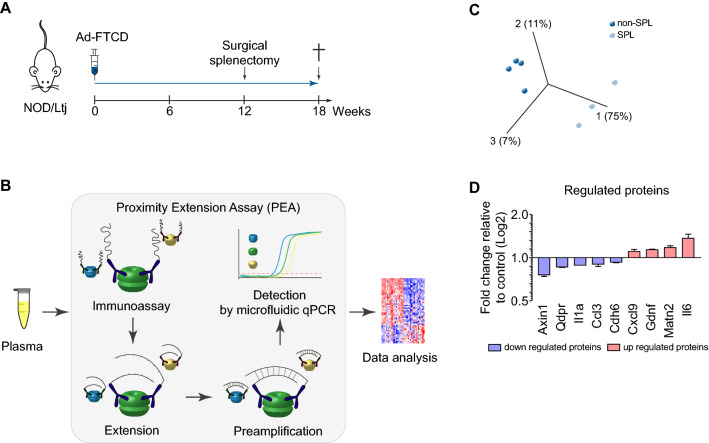
Serum protein signature shifts toward regeneration. **A** Experimental scheme of emAIH induction and splenectomy. **B** Scheme of Olink technology to quantify serum proteins. **C** Principal component analysis (PCA) of a dataset containing protein measurements in sera of splenectomized (*n* = 3) and non-splenectomized mice (*n* = 4) with emAIH. **D** Fold change (blue downregulated proteins, red upregulated proteins) of serum proteins 6 weeks after splenectomy

The pathogenesis and pathophysiology of AIH are still largely unknown. Likewise, the roles of different lymphocyte populations are still under discussion. Therefore, we induced emAIH with an adenovirus (Ad) encoding FTCD (Ad-FTCD) and removed the spleen at week twelve, which was during the late stage of the disease [9]. After 6 weeks, the mice were sacrificed (Fig. 1A). Serum samples were collected and analyzed, and we quantified 92 different proteins within them with Olink technology (Fig. 1B). In total, 9 of the proteins were differentially regulated with clustering within the two groups (Fig. 1C), with three exhibiting significant changes (*p* < 0.001) (Fig. 1D). Protein analyses demonstrated the downregulation of the inflammatory proteins chemokine (C–C motif) ligand 3 (CCL3), interleukin (IL)-1a and cadherin (Cdh)6 as well as Axin1 and quinoid dihydropteridine reductase (Qdpr). On the other hand, proteins related to regeneration such as Matrilin (Matn)-2, Glial cell-derived neurotrophic factor (Gdnf) and the myokine IL‐6 were upregulated. Notably, the chemokine Cxcl9 was upregulated and thought to be an inflammatory chemokine.

We also analyzed TH1-, TH2-, TH17- and fibrosis-related markers in liver tissue by quantitative PCR, but we could not find any differentially regulated genes in a set of 21 genes (data not shown).

In conclusion, splenectomy during the late stage of a chronic inflammatory disease shaped regeneration at the molecular level.

### Biochemical remission was induced by regenerative processes

We previously showed that splenectomy does not affect hepatic histology [1, 2]. Nonetheless, AIH is a chronic and progressive disease. Thus, the pathology at the histological and biochemical levels after 18 weeks was completely unknown with respect to the observed regenerative molecular pattern.

**Fig. 2:**
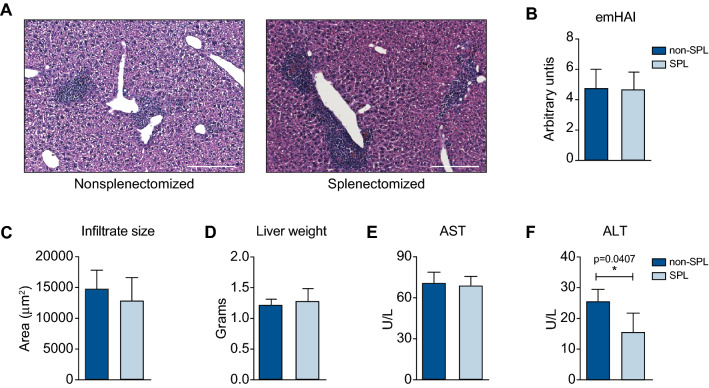
Biochemical remission was induced by regenerative processes. **A** Eighteen weeks after emAIH induction, hepatic sections of splenectomized (*n* = 5) and nonsplenectomized mice (*n* = 5) were taken to perform microscopic HE staining (**B**) and histological mHAI assessment. **C** Infiltrate sizes and **D** liver weights were measured. **E** Serum AST and **F** ALT levels were measured after splenectomy [splenectomized (SPL; *n* = 4), non-splenectomized (non-SPL; *n* = 4)]. Scale bars = 100 µm

Therefore, liver sections were produced and analyzed by microscopy. No obvious differences in histology could be observed at 6 weeks after splenectomy between splenectomized and non-splenectomized mice with emAIH (Fig. 2A). The same was true for the mHAI, which was higher than in comparison to the scores 6 weeks earlier from other studies [5, 9] but comparable between the two groups (Fig. 2B). Additionally, the average size of lymphatic infiltrates was just slightly smaller (Fig. 2C), while the liver weight was unchanged (Fig. 2D). However, biochemical analysis of the serum revealed a consequence of the regenerative molecular pattern observed. While the levels of aspartate transaminases (ASTs) were not prominently altered (Fig. 2E), the levels of alanine transaminases (ALTs) were significantly reduced by approximately 40% (Fig. 2F).

In conclusion, splenectomy induced biochemical remission of AIH.

### Increase in intrahepatic Tregs after splenectomy in the inflamed liver

The regenerative molecular pattern and biochemical remission of AIH after splenectomy were revealed, but the cellular mechanism remained unknown.

**Fig. 3:**
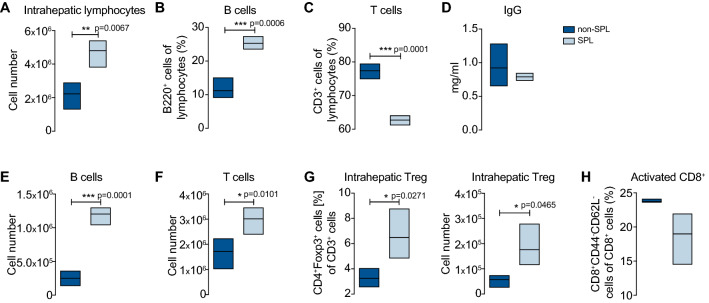
Increase in intrahepatic Tregs after splenectomy in the inflamed liver. **A** Splenectomized (SPL; *n* = 4) and non-splenectomized (non-SPL; *n* = 4) NOD/Ltj mice with emAIH were analyzed 6 weeks after splenectomy for quantification of total intrahepatic lymphocytes. **B** Flow cytometric analyses of B cells (**C**), T cells (**D**) and corresponding serum IgG levels. **E** B cells and (**F**) T cells were also quantified. **G** Flow-cytometric analyses of CD3^+^CD4^+^CD25^+^Foxp3^+^ Tregs as well as their quantification of total Tregs in the liver. **H** Percentage of intrahepatic activated CD3^+^CD8^+^ effector T cells

Therefore, we analyzed the cellular composition of IHLs. In contrast to the changes observed in other models, the removal of the spleen, which resulted in the loss of a lymphocyte site, was compensated by an increase in lymphocytes in the liver (Fig. 3A). Here, the relative numbers of B cells were increased after splenectomy (Fig. 3B). Consequently, the relative number of T cells decreased (Fig. 3C). The IgG serum level did not subsequently change (Fig. 3D).

Given that the spleen was removed, the increase in B cells (Fig. 3E) and T cells in absolute numbers was not surprising as the liver is a harbor for lymphocytes (Fig. 3F). To investigate the outcome of the regenerative serum protein pattern and cause of biochemical remission, other cells were evaluated. Analyses revealed that both the relative and absolute numbers of intrahepatic Tregs were increased (Fig. 3G). This supraproportional increase even within the enlarged T-cell compartment of the immune regulatory and tissue-repairing cell subpopulation was meaningful. Consequently, the number of activated CD8^+^ T cells was decreased (Fig. 3H). In summary, the most prominent immunoregulatory cell population was supraproportionally increased in the liver.

**Fig. 4:**
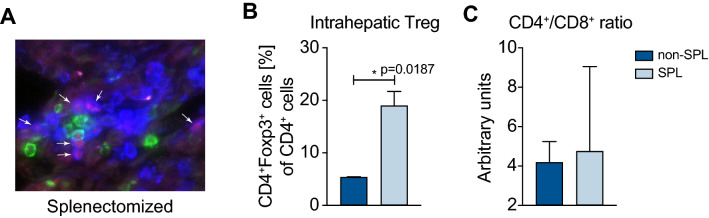
Local increase in intrahepatic Tregs after splenectomy. Splenectomy induced excessive numbers of local Tregs in liver tissue (**A**) Cryo-preserved liver sections were immunohistochemically stained for CD4 (blue), CD8 (green) and Foxp3 (red). **B** The staining was quantified, as were the ratio of Tregs among CD4^+^ cells, (C) and the ratios of CD4^+^ and CD8^+^ T cells in splenectomized (*n* = 5) and non-splenectomized mice (*n* = 5)

In addition to all intrahepatic lymphocytes, we analyzed the local population within the portal inflammation. For this purpose, we prepared cryosections from liver tissue. To reduce the immense background, the organs were treated with sucrose before embedding. Frozen organs were sectioned to 4 µm and stained for CD4, CD8 and Foxp3 (Fig. 4A). CD4 (blue) T cells with Foxp3 (red) within the nuclei are easily visible, which are Tregs. In the animals with emAIH that were additionally splenectomized, there was an even more significant increase in Tregs than in the flow cytometric analysis of all IHLs (Fig. 4B).

### An increase in intrahepatic Tregs correlates with a reduction in ALT

Given that the CD4/CD8 ration is decreased in other liver diseases if the disease is worsening, we analyzed this in our splenectomy emAIH model.

**Fig. 5:**
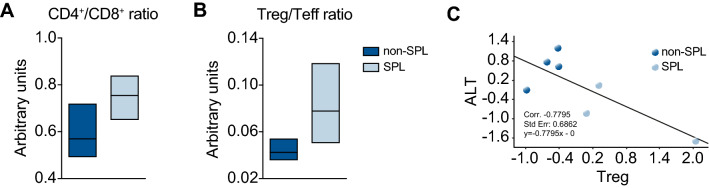
An increase in intrahepatic Tregs correlates with reduced ALT (**A**) Ratios of CD4^+^ to CD8^+^ T cells and (**B**) Tregs to T effector cells (Teff) within the IHLs. **C** Correlation analyses of ALT versus Tregs 6 weeks after splenectomy

As in other models and diseases, the ratio of CD4^+^ to CD8^+^ T cells was slightly increased within the shrunken T-cell compartment (Figs. 4C and 5A). The same was true for the ratio of Tregs to Teffs (Fig. 5B). We also noted a slight increase in the Treg/Teff ratio, but this increase was not significant due to the normal error distribution. In contrast, the correlation analysis of Tregs and ALT showed a good correlation between an increase in Tregs and a decrease in ALT (Fig. 5C).

The observed increase in intrahepatic Tregs might have been the cause of the observed biochemical remission.

## Discussion

We showed that 6 weeks after splenectomy there was an increase in intrahepatic Tregs and a decrease in activated CD8^+^ T cells. Consequently, biochemical remission of emAIH was characterized by improvements in transaminase levels. This remission was accompanied by a shift in the signature of serum proteins toward regeneration. The increase in intrahepatic Tregs and the improvement in ALT levels showed a good correlation, suggesting causality. At the cellular level, there was also an imbalance in the ratio between T cells and B cells, with a disproportionate increase in the total number of B cells.

In the 1960s, small cohorts of patients with chronic aggressive hepatitis were treated by splenectomy in Germany [17, 18] and Romania [24]. While some patients benefitted from this approach, others died. Notably, in 1970, hepatitis B virus was discovered. Thus, AIH and viral hepatitis could not be discriminated in the 1960s. One could assume that the patients who benefited from this therapy did not suffer from viral hepatitis. It is therefore of great interest to determine which mechanisms underlay this positive effect.

However, we did not observe the effects of long-term injury after splenectomy at the molecular level, such as increased IL-1a or TNF-α. Overall, a more regenerative molecular pattern has not been described before in AIH. In contrast, before the development of AIH, we and others have described and postulated a protective role for the spleen in autoimmune hepatitis [1, 25], even if its role in the etiology of AIH is less important [2–4]. We demonstrated here that this protective effect is not observed in the late phase of established disease.

As mentioned previously, the numbers of intrahepatic T cells were reduced, as shown by flow cytometry data, but within this smaller T-cell population, Tregs were increased in number and proportion in the inflamed liver tissue. This has also been previously described for hepatic inflammatory conditions such as autoimmune diseases and graft rejection in patients under therapy [26–28]. Similarly, this mechanism of regeneration was more pronounced in our model after splenectomy. One could speculate about the causality of the increased Tregs and the regenerative proteome. However, we suggest that these molecular factors are precursors to a cellular response.

The less-activated/more-naïve phenotype of CD8^+^ T cells is a clear indication of immune regulation. Moreover, activated T cells are needed in most models of autoimmunity in the liver [29–33]. Similarly, others have demonstrated a mechanism by which cross-priming by LSECs results in tolerance in CD8^+^ T cells [34]. In contrast, the roles of the B-cell population and increased IgG and autoantibody levels are still not understood [16, 35–37]. This is particularly noteworthy because the different autoantibody patterns have high diagnostic value and because depletion of B cells improves the course of disease in AIH models [10, 11, 16, 37]. Unfortunately, the role of B cells could not be determined in this model of splenectomy in emAIH.

## Conclusions

In conclusion, biochemical remission of emAIH was induced by splenectomy performed during the late stage of disease. This remission was caused by two regenerative mechanisms. On the one hand, we observed marked enrichment of intrahepatic Tregs; on the other hand, we observed a molecular signature of serum proteins that was clearly consistent with the hallmark of regeneration.

## Data Availability

The data presented in this study are available on request from the corresponding author.
